# The Keystone Veterinary Medical Association

**Published:** 1890-12

**Authors:** C. H. Magill

**Affiliations:** Secretary


*The Keystone Veterinary Medical Association*—The Keystone Veterinary Medical Association held its monthly meeting at the College of Physicians, Philadelphia, on November 1st, the president, Dr. Hopkins, in the chair. A communication was read from Dr. W. B. E. Miller explaining his absence from meetings and his failure to present a paper. Present: Drs. Hoskins, Weber, Kooker, Bridge, Zuill, Williams, Lintz, Cullen, Eves, Harger, Formad, Magill and Mattson. The trustees reported favorably the names of Drs. Landis and Drake for membership. They were elected and presented to the Society. Dr. Hoskins, for the legislative committe, reported that the prothonotary of Philadelphia County was doing his duty in regard to the registration of veterinarians. Dr. Bridge reported that 400 hogs suffering from hog cholera had been shipped from the Western part of the State to the markets of Pittsburgh, Philadelphia and New York. Four cases of glanders, one in Colmar, one in West Philadelphia and two in Stroudsburg, had been killed and the owners indemnified by the State, at $25 per horse.

Dr. Zuill suggested that he believed it would be to the interest of the Association to have fewer business meetings and more devoted to scientific work. His remarks were favorably commented upon by Drs. Bridge and Weber. The president requested him to place his suggestion in the shape of a resolution. The president read a communication from Dr. Zuill, making charges against Dr. Huidekoper, for violation of the code of ethics, in issuing a circular, stating that he had accepted a chair in the American Veterinary College and had opened an office in New York, and for stating on his card, "examination for soundness and diseases of dogs a specialty." It was referred to the Board of Trustees.

The president called Dr. Kooker to the chair, and taking the floor, offered the following resolutions:

WHEREAS, The Veterinary Department of the University of Pennsylvania have publicly announced in an appeal through the *Press*, that hereafter veterinary treatment on their part will be free to all who apply for the same.

Inasmuch as we are compelled to earn our living through such services, it seems an unfair advantage for that institution to resort to such means as our competitor.

With the best facilities in the land and a location unsurpassed, and with power to command the greatest talent of our land or the English-speaking world, such a step on their part should meet with our just censure and deprecation, and place that institution entirely outside the pale of our recognition and support.

And we do further appeal to them to rescind this declaration.

(Signed) W. HORACE HOSKINS.

Remarks on the subject favorable to the resolutions were made by Drs. Hoskins, Lintz, Eves, Cullen, Weber, Bridge and Kooker. The action of the College was defended by Drs. Zuill and Harger. A vote on the resolutions was called for and it was adopted. Ayes—Hoskins, Drake, Cullen, Eves, Weber, Bridge, Mattson and Lintz; nays—Zuill, Harger, Landis, Formad and Magill.

The following resolution was offered:

Moved that the chair appoint a committee of three whose duty it shall be to choose a subject for debate at each meeting, and that the President appoint at least two debaters on each side of the question, and that the committee be continued for this work.

(Signed) W. L. ZUILL,

F. BRIDGE.

Also;

I move that the business meetings of this association be limited to four meetings per year.

(Signed) W. L. ZUILL,

F. BRIDGE.

The President appointed Drs. Zuill, Bridge, and Kooker a committee.

Dr. Vandergrift, the essayist, was absent. Dr. Mattson reported a case of a cow having twins eight days apart. Dr. Bridge reported one having twins two days apart. Dr. Mattson reported a case of torsion of uterus. Dr. Bridge was asked by Dr. Zuill to report a case of glanders in a cow at the next meeting.

Dr Kooker reported a case of suppurated foot affecting the tendons and joint and which had to be destroyed.

Dr. Kooker asked for opinions as to the best kind of treatment for the same. Dr. Eves advised amputation. Dr. Zuill advised amputation only in mare in foal, but, in other cases, advised street nail operation.

Dr. M. W. Drake reported a case of pyæmia, due to encysted abscess, as follows: A bay gelding, six years old, found atanding with all four feet close together under the body, very much drawn up, could move forward but not backward, a staring countenance, eyes bulging and glassy, no feeling in them but sight good. Temperature, 105. Pulse, 56. Respiration about normal. Urine and feces normal, taking feed and water in the usual quantities, ate greedily, gradually regained his normal standing position, continued in about the same condition for five days, beginning to favor off hind leg, then laid down unable to rise, got him up, off hind leg very much swollen below the hock, not hot or painful to the touch, respirations inereasing, saw him next day about 4 P. M., an enormous slough had taken place just above and on the inside of the fetlock, discharging large quantities of blood and synovia without fœter or smell, exposing both flexors, tendons, and suspensory ligament, respirations quickened, otherwise, condition unchanged.

Seen again on the eighth day, found a dark-brown discharge from both nostrils, accompanied by a gangreous smell, the flexor tendons entirely seperated, animal very dull and weak, still eating his feed regularly, ordered destroyed, died next morning at 6 A. M.

*Post-Mortem.*—The blood clotted like jelly, very hard and black, both lungs gangrenous, a large abscess in anterior lobe of left lung, of a grayish-white color, broken down to a liquid state, and nearly empty, the surrounding parts of the lung were filled with this material, soft and friable. Two abscesses in the right lung as large as an egg, consisting of hard, yellow, cheesey mass, completely encysted.

Heart filled with dark, clotted blood, very much enlarged and softened.

The left kidney, found in an enlarged light, puffy condition, its strucsure entirely masked.

The right kidney, beginning to enlarge and soften on its external portions.

The liver very dark colored and softened.

The spleen, stomach, and intestines normal.

Meeting then adjourned.

C. H. MAGILL, *Secretary.*

